# Rotational Atherectomy and Stent Implantation for Calcified Left Main Lesions

**DOI:** 10.4021/cr78w

**Published:** 2011-09-20

**Authors:** Bryan G. Schwartz, Guy S. Mayeda, Christina Economides, Robert A. Kloner, David M. Shavelle, Steven Burstein

**Affiliations:** a Heart Institute, Good Samaritan Hospital, Los Angeles, California, USA; b Department of Cardiology, Good Samaritan Hospital, Los Angeles, California, USA; c Department of Internal Medicine, Division of Cardiovascular Medicine, Keck School of Medicine at the University of Southern California, Los Angeles, CA, USA

**Keywords:** Cardiac catheterization, Drug-eluting stent, Devices

## Abstract

**Background:**

Left main coronary artery (LMCA) bifurcation and heavily calcified lesions are common and challenging to treat percutaneously. Rotational atherectomy (RA) may be beneficial in this setting to facilitate stent placement though direct supporting evidence is lacking. This study sought to analyze patients who underwent RA of the LMCA.

**Methods:**

Consecutive cases involving RA of the LMCA between 1/1/2004 and 12/31/2009 at a private, tertiary referral hospital were reviewed retrospectively. Medical records, angiograms and clinically driven follow-up were reviewed.

**Results:**

Thirty-one cases were identified (20 protected, 11 unprotected), including 23 with stent implantation (21 drug-eluting, 2 bare metal). All 31 lesions had moderate to severe calcification, 84% involved the distal segment. Mean burr-to-vessel ratio was 0.43. Overall angiographic success was 90% (28/31) and was higher with a drug-eluting stent versus no stent (100% vs. 62%; P = 0.0153). In-hospital major adverse cardiovascular events (MACE) occurred in 1 patient (3%). Mid-term MACE occurred in 6 patients (26%) and tended to occur less frequently in patients with protected LMCAs (P = 0.0697). At final follow-up, patients were more likely to be alive and free from angina with a protected LMCA (94% vs. 57% unprotected; P = 0.0564) and with a drug-eluting stent (89% vs. 50% with no stent; P = 0.0281).

**Conclusions:**

RA of the LMCA to facilitate stent implantation appears to be safe and effective with favorable mid-term outcomes. In the setting of severe calcification and distal LMCA involvement RA and drug-eluting stent implantation should be considered.

## Introduction

Percutaneous coronary intervention (PCI) is often performed on patients with significant disease in a protected (left coronary grafted) left main coronary artery (LMCA). For selected patients with unprotected LMCA disease, recent evidence regarding PCI with drug-eluting stent (DES) implantation suggests a benefit similar to coronary artery bypass graft (CABG) surgery, although this approach is highly debated [1, 2]. For patients with unprotected LMCA disease who are not eligible for CABG, PCI currently has a class II indication [3]. Certain LMCA lesion subsets remain especially challenging for PCI, including the distal bifurcation and heavily calcified lesions [2, 4, 5]. The distal bifurcation is involved in over 60% of unprotected LMCA lesions and predicts a worse clinical outcome [2, 4, 5]. Alternative interventions, such as the use of debulking devices, may improve the endovascular management of these lesion subsets.

Rotational atherectomy (RA) utilizes a diamond coated burr that rotates at high speed (120,000 - 200,000 revolutions per minute) to ablate atherosclerotic plaque [6]. In heavily calcified lesions, RA facilitates stent implantation and improves acute gain [7-11]. Also, RA preserves the patency of side branches in bifurcation and ostial lesions [12, 13]. RA alone or with bare metal stents resulted in unacceptably high restenosis rates; however, at 6 months to 3 years of follow-up RA with DESs resulted in target lesion revascularization rates ranging from 2% to 10.6% [14-18]. Reports of RA involving the LMCA are scarce [19]. We sought to analyze procedural characteristics and clinical outcomes in a group of patients that underwent RA to facilitate DES implantation in the LMCA to better define the use of RA in the LMCA in contemporary practice.

## Methods

Approval was obtained from the Western Institutional Review Board. For this retrospective analysis, the cardiac catheterization database at a private, tertiary referral hospital was searched to identify all cases involving RA in the LMCA between 1/1/2004 and 12/31/2009. A comprehensive chart review was conducted to record pertinent data on each case, including demographics, medical history, procedural characteristics, hospital course, and mid-term follow-up. Angiograms were reviewed and quantitative coronary angiography (QCA) was performed to define lesion and interventional characteristics.

### Definitions

Lesion calcification was defined prior to contrast injection as follows: severe if radiopacities were readily apparent without cardiac motion, moderate if radiopacities were apparent only with cardiac motion, mild if faint radiopacities were seen only with cardiac motion, or none if no radiopacities were apparent [20]. A lesion was considered bifurcating if a branch (> 1.5 mm) originated within the stenosis, the branch had ostial disease, and the branch was completely surrounded by stenotic portions within the parent vessel lesion [20]. Clinical and angiographic follow-up were clinically driven, not mandated. Clinical follow-up was only included for patients that returned to our facility and were evaluated by our physicians. Major adverse cardiac events (MACE) were defined as death, Q-wave myocardial infarction, target vessel revascularization, or new creatine kinase elevation above 2 times the upper limit of normal post-PCI. Angiographic success was defined as < 40% residual stenosis and TIMI grade 3 flow at the conclusion of the procedure. Procedural success was defined as angiographic success in the absence of MACE. Target vessel revascularization was defined as PCI or CABG to treat restenosis within the LMCA. Complications occurring during the index hospitalization were classified as acute, and complications occurring after hospital discharge were considered mid-term (mean 11.4 months; range 0 - 54 months). Binary restenosis was defined as > 50% diameter stenosis of the target lesion at follow-up angiography.

### Treatment

All medical decisions, including medications and PCI, were at the discretion of the interventionalist. RA was used primarily to change the compliance of a calcified artery (30 of 31, 97%) and to prevent side branch occlusion (16 of 31, 52%) (Table 1). In 2 patients (6%) RA was not planned but was used after attempts to deliver a balloon had failed. The final burr-to-vessel ratio was 0.43 ± 0.07.

**Table 1:** Procedural Characteristics

	Total	Protected	Unprotected	P-value	No Stent	DES	P-value
Reason for RA							
Outright/planned	94% (29/31)	90% (18/20)	100% (11/11)		100% (8/8)	95% (20/21)	
Moderate or severe calcification	97% (30/31)	95% (19/20)	100% (11/11)		100% (8/8)	95% (20/21)	
Prevent side branch occlusion	52% (16/31)	45% (9/20)	64% (7/11)		38% (3/8)	62% (13/21)	
Decrease lesion eccentricity	23% (7/31)	35% (7/20)	0% (0/11)		12% (1/8)	24% (5/21)	
In-stent restenosis	3% (1/31)	0% (0/20)	9% (1/11)		12% (1/8)	0% (0/21)	
Bail-out/balloon did not pass	6% (2/31)	10% (2/20)	0% (0/11)	0.527	0% (0/8)	5% (1/21)	1.0
Burrs							
Number of burrs	1.3 ± 0.5 (21)	1.2 ± 0.4 (20)	1.4 ± 0.7 (11)	0.595	1.4 ± 0.7 (8)	1.2 ± 0.4 (21)	0.378
Final burr size (mm)	1.51 ± 0.22 (21)	1.50 ± 0.21 (20)	1.54 ± 0.24 (11)	0.689	1.52 ± 0.31 (8)	1.50 ± 0.16 (21)	0.101
Final burr-to-vessel ratio	0.43 ± 0.07 (21)	0.44 ± 0.08 (20)	0.41 ± 0.06 (11)	0.132	0.43 ± 0.06 (8)	0.43 ± 0.06 (21)	0.877

Numbers represent mean ± standard deviation or a percentage of the total.

BMS = bare metal stent; DES = drug-eluting stent; RA = rotational atherectomy; atm = atmospheres.

Balloon angioplasty was used in 26 cases (84%) with a mean maximum inflation pressure of 9.1 ± 4.9atm. Stents were implanted in 23 lesions (81%), 21 of which were DESs (12 Taxus, Boston Scientific, Natick, MA, USA; 6 Cypher, Cordis, Bridgewater, NJ, USA; 3 other). The 2 bare metal stents (Multi-Link Vision, Abbott Vascular, Abbott Park, IL, USA) were used because of a history of stent thrombosis in 1 case, and impending surgery for bladder cancer in another. Stents were not used in 8 cases due to vessel size mismatch (3 patients), a good result after RA (3 patients) and to avoid jailing a major branch (2 patients). In this institution, when DESs are implanted in an unprotected LMCA, clopidogrel is continued indefinitely unless the risk of bleeding becomes too high.

Procedure times were long (mean 99 ± 24 min) and contrast use was high (mean 208 ± 86 ml). Utilization of intravascular ultrasound was low (4/31; 13%). Eight of 11 (73%) procedures on unprotected LMCAs were performed with a prophylactic left ventricular assist device (in 7 cases TandemHeart™, Cardiac Assist, Inc, Pittsburgh, PA, USA). All patients treated with prophylactic left ventricular assist devices had an ejection fraction ≤ 40% (mean 28±10%).

### Quantitative coronary angiography

QCA was performed by an experienced angiographer using MDQM-QCA (Medcon Quantitative Measurements – Quantitative Coronary Arteriography, Medcon Limited, Tel Aviv, Israel) edge-detection software. For each image calibration was done by performing QCA on a segment of catheter with known diameter. All QCA measurements were confined to the LMCA only, even if the intervention also involved another coronary artery (i.e. ostial circumflex). The images with the least amount of foreshortening and the highest degree of stenosis were selected for analysis. Lesion length was determined using the pre-intervention angiogram with the least amount of foreshortening. Reference vessel diameter was determined using the final image (post-intervention) at the angiographically normal-appearing proximal LMCA (for proximal lesions the segment with the largest diameter was used). Minimal luminal diameter (MLD) was determined up to four times: 1) pre-intervention, 2) post-RA (and balloon angioplasty if balloon angioplasty was done), 3) post-stent (if a stent was placed), and 4) at follow-up (if available). The % diameter stenosis at each instance was determined by dividing the MLD at that instance by the reference vessel diameter. Acute gain was defined as post-intervention MLD minus pre-intervention MLD. Late loss was defined as post-intervention MLD minus follow-up MLD.

### Statistics

Results are reported as the mean ± standard deviation or percentages of the total. Patients with protected LMCAs were compared with patients with unprotected LMCAs. Patients treated with a DES were compared with patients treated without a stent (patients treated with a BMS were excluded from this comparison). Student’s t-test was used to compare continuous variables. Fischer’s exact test was used to compare class variables (SAS 9.1, Cary, NC). Kaplan-Meier estimates and log-rank tests were performed using the R statistical software system, Ver:2.11.1 (www.r-project.org). Statistical significance was considered a P-value < 0.05.

## Results

A total of 31 patients underwent RA of the LMCA, including 20 with protected and 11 with unprotected lesions (Table 2). Twenty-one patients received a DES, 2 received a bare metal stent and 8 patients did not receive a stent. Patients were elderly (mean 75.5 ± 9.4 years) with longstanding coronary artery disease (68% had a history of CABG at a mean of 10.0 years prior). Medical comorbidities included hypertension in 84%, hyperlipidemia in 65% and diabetes mellitus in 61%. In addition to left main disease, 26 of 31 patients (84%) also had 3 vessel coronary disease and many patients had severe comorbidities and poor functional status. Baseline characteristics were similar between groups, except that patients undergoing unprotected LMCA interventions had lower ejection fractions than those with protected LMCAs. The indications for PCI of the LMCA in the 11 patients with unprotected disease included severe comorbidities (5 patients), poor functional status (5 patients), advanced age (4 patients), and poor targets (2 patients) (some patients had multiple reasons). Patients undergoing interventions on protected LMCAs tended to present with angina, whereas a recent myocardial infarction was more common in patients with unprotected LMCAs.

**Table 2:** Patient Demographics, Medical History and Presentation

	Total	Protected	Unprotected	P-value	No Stent	DES	P-value
Number of patients	31	20	11		8	21	
Age, mean ± SD (n) (years)	75.5 ± 9.4 (31)	74.8 ± 8.4 (20)	76.9 ± 11 (11)	0.560	74.6 ± 9.7 (8)	76.8 ± 8.8 (21)	0.565
Gender, % (n) male	71% (22/31)	75% (14/20)	73% (8/11)	1.0	75% (6/8)	71% (15/21)	1.0
Race, % (n) Caucasian	32% (10/31)	38% (8/20)	18% (2/11)	0.437	25% (2/8)	33% (7/21)	0.954
% (n) Asian	26% (8/31)	25% (4/20)	36% (4/11)		25% (2/8)	29% (6/21)	
% (n) Hispanic	32% (10/31)	25% (5/20)	45% (5/11)		38% (3/8)	29% (6/21)	
% (n) African-American	6% (2/31)	12% (2/20)	0% (0/11)		12% (1/8)	5% (1/21)	
Hypertension	84% (26/31)	88% (18/20)	73% (8/11)	0.317	100% (8/8)	76% (16/21)	0.283
Diabetes mellitus	61% (19/31)	55% (11/20)	73% (8/11)	0.452	50% (4/8)	62% (13/21)	0.683
Insulin-dependent	19% (6/31)	10% (2/20)	36% (4/11)	0.151	12% (1/8)	24% (5/21)	0.647
Hyperlipidemia	65% (20/31)	85% (17/20)	27% (3/11)	0.004	62% (5/8)	67% (14/21)	1.0
Current smoker	6% (2/31)	5% (1/20)	9% (1/11)	1.0	0% (0/8)	5% (1/21)	1.0
Prior PCI	32% (10/31)	30% (6/20)	36% (4/11)	1.0	25% (2/8)	38% (8/21)	0.675
Remote MI	26% (8/31)	30% (6/20)	18% (2/11)	0.676	38% (3/8)	19% (4/21)	0.357
Prior CABG	68% (21/31)	100% (20/20)	9% (1/11)	< 0.001	62% (5/8)	67% (14/21)	1.0
Years since CABG	10.0 ± 5.8 (21)	10.4 ± 5.7 (20)	3 (1)		8 ± 7 (8)	11 ± 5 (21)	
Creatinine (mg/dl)	1.5 ± 1.1 (31)	1.4 ± 1.2 (20)	1.5 ± 0.9 (11)	0.861	1.1 ± 1.3 (8)	1.4 ± 0.6 (21)	0.368
% (n) ≥ 1.4	32% (10/31)	30% (6/20)	36% (4/11)	1.0	25% (2/8)	38% (8/21)	0.372
Hemodialysis	5% (2/31)	5% (1/20)	9% (1/11)	1.0	0% (0/8)	5% (1/21)	1.0
Ejection fraction	44±15% (30)	49±13% (19)	34±14% (11)	0.004	41±14% (8)	44±15% (21)	0.618
% (n) ≤ 50%	70% (21/30)	58% (11/19)	91% (10/11)	0.1	88% (7/8)	62% (13/21)	0.372
Presentation				0.051			0.145
Stable angina	45% (14/31)	50% (10/20)	36% (4/11)		50% (4/8)	48% (10/21)	
Unstable angina	16% (5/31)	25% (5/20)	0% (0/11)		0% (0/8)	24% (5/21)	
Silent ischemia	13% (4/31)	15% (3/20)	9% (1/11)		0% (0/8)	14% (3/21)	
Recent MI	23% (7/31)	10% (2/20)	45% (5/11)		38% (3/8)	14% (3/21)	
Cardiogenic shock	3% (1/31)	0% (0/20)	9% (1/11)		12% (1/8)	0% (0/21)	
LMCA + 2 vessel disease	13% (4/31)	0% (0/20)	36% (4/11)	0.010	12% (1/8)	14% (3/21)	1.0
LMCA + 3 vessel disease	84% (26/31)	95% (19/20)	64% (7/11)	0.042	88% (7/8)	81% (17/21)	1.0
Moderate-severe calcification	100% (31/31)	100% (20/20)	100% (11/11)	1.0	100% (8/8)	100% (21/21)	1.0
Involved distal segment	84% (26/31)	85% (17/20)	82% (9/11)	1.0	75% (6/8)	86% (18/21)	0.597
Involved bifurcation	65% (20/31)	70% (14/20)	55% (6/11)	0.452	62% (5/8)	62% (13/21)	1.0

Numbers represent mean ± standard deviation or a percentage of the total.

CABG = coronary artery bypass graft surgery; DES = drug-eluting stent; MI = myocardial infarction; PCI = percutaneous coronary intervention.

All LMCA lesions were moderately (3 lesions; 10%) or severely (28 lesions; 90%) calcified. Most lesions involved the distal segment of the LMCA (26 of 31; 84%) and the bifurcation (20 of 31; 65%) (some distal LMCA lesions with totally occluded branches were not bifurcation lesions). Interventions on protected LMCAs more often involved the ostial left circumflex coronary artery (14 of 20 patients, 70%). Conversely, interventions on unprotected LMCAs tended to involve the ostial left anterior descending coronary artery (7 of 11 patients, 64%).

Angiographic success was achieved in 28 of the 31 patients (90%), and procedural success in 27 of 31 (87%) (Table 3). In patients without a reason to avoid a DES, RA successfully facilitated DES implantation in all cases (21 of 21 patients, 100%) despite severe calcification. All PCIs with DES implantation achieved angiographic success (21 of 21; 100%). Angiographic success was significantly more likely in patients treated with a DES compared with no stent (100% vs. 62%; P = 0.0153). All 3 patients without angiographic success (because of residual diameter stenoses of 42-53%) had a contraindication to CABG surgery and a reason to avoid a stent.

**Table 3:** Quantitative Coronary Angiography

	Total	Protected	Unprotected	P-value	No Sent	DES	P-value
Lesion length (mm)	6.55 ± 3.0 (31)	6.5 ± 3.2 (20)	6.6 ± 2.7 (11)	0.905	6.6 ± 3.8 (8)	6.6 ± 2.9 (21)	0.967
Reference vessel diameter	3.55 ± 0.50 (31)	3.41 ± 0.43 (20)	3.80 ± 0.52 (11)	0.031	3.57 ± 0.63 (8)	3.51 ± 0.43 (21)	0.796
Initial MLD	1.18 ± 0.59 (31)	1.01 ± 0.54 (20)	1.50 ± 0.57 (11)	0.026	1.20 ± 0.79 (8)	1.14 ± 0.52 (21)	0.827
% Diameter stenosis							
Initially	67±15% (31)	70±15% (20)	61±13% (11)	0.076	68±18% (8)	67±14% (21)	0.933
After RA ± PTCA	40±13% (31)	39±14% (20)	41±12% (11)	0.708	34±17% (8)	42±11% (21)	0.145
After stent	14±12% (23)	15±13% (16)	11±12% (7)	0.487		13±13% (21)	
At follow-up	20±18% (11)	19±19% (9)	25±18% (2)		38% (1)	18±19% (9)	
Acute gain							
With no stent	1.11 ± 0.76 (8)	1.55 ± 0.71 (4)	0.67 ± 0.58 (4)		1.11 ± 0.76 (8)		
With stenting	1.84 ± 0.64 (23)	1.85 ± 0.74 (16)	1.82 ± 0.36 (7)			1.87 ± 0.66 (21)	
Follow-up angiography available	35% (11/31)	45% (9/20)	18% (2/11)		12% (1/8)	43% (9/21)	
Mean ± SD (n) (months)	15.8 ± 11.6 (11)	15.5 ± 12.3 (9)	17.2 ± 10.9 (2)		25.0 (1)	13.7 ± 11.8 (9)	
Late loss (mm)	0.26 ± 0.40 (11)	0.24 ± 0.44 (8)	0.32 ± 0.18 (2)		0.19 (1)	0.27 ± 0.45 (9)	
Binary restenosis	9% (1/11)	11% (1/9)	0% (0/2)		0% (0/1)	11% (1/9)	
Angiographic success	90% (28/31)	95% (19/20)	82% (9/11)	0.281	62% (5/8)	100% (21/21)	0.015
Procedural success	87% (27/31)	95% (19/20)	73% (8/11)	0.115	62% (5/8)	95% (20/21)	0.052

Numbers represent mean ± standard deviation or a percentage of the total.

DES = drug-eluting stent; MLD = minimal luminal diameter; PTCA = percutaneous transluminal coronary angioplasty; RA = rotational atherectomy.

There were no procedural complications (Table 4). Acute MACE occurred in 1 of 31 patients (3%); one 97-year-old patient with an unprotected LMCA, dementia and multiple comorbidities died of pneumonia, renal failure and congestive heart failure 14 days after the procedure.

**Table 4:** In-Hospital Outcomes and Procedural Complications

	Total
Death	3% (1/31)
CABG	0% (0/31)
Q-wave MI	0% (0/31)
Non Q-wave MI	0% (0/31)
MACE	3% (1/31)
Dissection	0% (0/31)
Perforation	0% (0/31)
Spasm	0% (0/31)
Thrombosis	0% (0/31)
No reflow	0% (0/31)
Side branch occlusion	0% (0/31)
Hypotension and/or bradycardia	0% (0/31)

Numbers represent a percentage of the total.

* = 1 death was unprotected LMCA treated with a drug-eluting stent; CABG = coronary artery bypass graft surgery; MACE = major adverse cardiovascular events; MI = myocardial infarction.

The results of QCA are shown in Table 3. Acute gain after RA ± balloon angioplasty was 0.93 ± 0.62 mm. Total acute gain for the 23 patients with stent implantation was 1.84 ± 0.64 mm. A mean initial percent diameter stenosis of 67±14% was reduced to a mean of 13±13% after RA and DES implantation.

**Figure 1:**
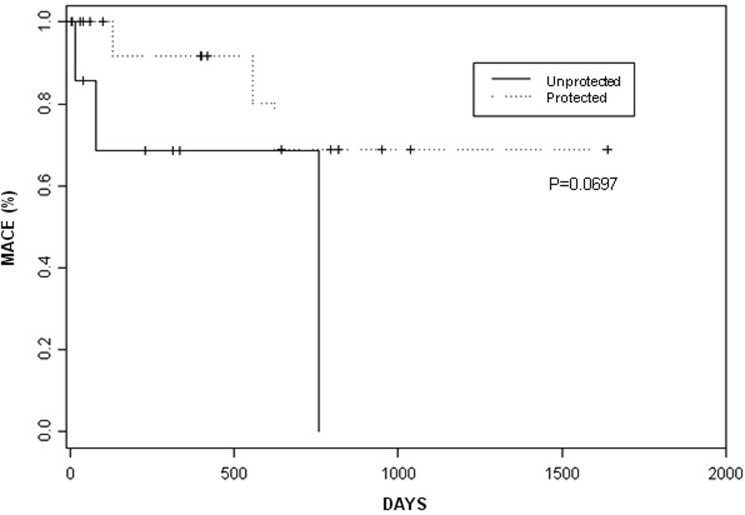
Major adverse cardiovascular events in patients with protected and unprotected left main coronary artery interventions. Kaplan-Meier incidence curves of major adverse cardiovascular events (MACE) in patients with protected and unprotected left main coronary artery interventions. Incidences in the 2 groups were statistically compared with a log-rank test.

Clinical follow-up was available in 74% of all patients and in 86% of the DES patients at a mean of 15.3 ± 14.7 months (Table 5). Overall, MACE occurred in 6 patients (26%) at 11.9 ± 10.6 months (1 non-ST elevation myocardial infarction, 2 target vessel revascularizations, 3 deaths). During follow-up, MACE tended to occur less frequently in patients with protected LMCAs vs. unprotected LMCAs (P = 0.0697 by log-rank; Fig. 1). No differences were observed in MACE rates or death rates in patients treated with a DES compared with no stent.

**Table 5:** Mid-term Outcomes

	Total	Protected	Unprotected	P-value	No Stent	DES	P-value
Follow-up available	74% (23/31)	80% (16/20)	64% (7/11)	0.405	50% (4/8)	86% (18/21)	0.068
Mean ± SD (n) (months)	15.3 ± 13.9 (23)	18.4 ± 14.8 (16)	8.3 ± 8.5 (7)		13.0 ± 11.9 (4)	15.3 ± 14.7 (18)	
MACE	26% (6/23)	19% (3/16)	43% (3/7)	0.638†	50% (2/4)	22% (4/18)	1.0
Mean ± SD (n) (months)	11.9 ± 10.6 (6)	14.3 ± 8.8 (3)	9.3 ± 13.6 (3)		14.7 ± 14.7 (2)	10.5 ± 10.4 (4)	
Death	13% (3/23)	6% (1/16)	29% (2/7)	0.281	25% (1/4)	11% (2/18)	1.0
Mean ± SD (n) (months)	2.4 ± 1.9 (3)	4.3 (1)	1.5 ± 1.5 (2)		4.3 (1)	1.5 ± 1.5 (2)	
CABG	0% (0/22)						
Q-wave MI	0% (0/22)						
Non Q-wave MI	4% (1/23)	0% (0/16)	14% (1/7)	0.355	25% (1/4)	0% (0/18)	0.276
Mean (n) (months)	25.0 (1)		25.0 (1)		25.0 (1)		
Target vessel revascularization*	9% (2/23)	9% (2/23)	0% (0/7)	1.0	0% (0/4)	9% (2/23)	1.0
Mean (n) (months)	19.4 ± 1.6 (2)	19.4 ± 1.6 (2)					
Recurrent angina	18% (4/22)	19% (3/16)	17% (1/6)	1.0	50% (2/4)	12% (2/17)	0.555
Mean ± SD (n) (months)	17.0 ± 8.9 (4)	14.3 ± 8.8 (3)	24.9 (1)		14.6 ± 14.7 (2)	19.4 ± 1.6 (2)	
Alive and free from angina	83% (19/23)	94% (15/16)	57% (4/7)	0.056	50% (2/4)	89% (16/18)	0.028
Mean ± SD (n) (months)	16.8 ± 14.1 (19)	19.3 ± 14.8 (15)	7.6 ± 4.6 (4)		11.3 ± 14.1 (2)	17.5 ± 14.4 (16)	

Numbers represent mean ± standard deviation or a percentage of the total.

* = 1 target lesion revascularization; † = P-value is 0.0697 by log-rank test; CABG = coronary artery bypass graft surgery; DES = drug-eluting stent; LMCA = left main coronary artery; MACE = major adverse cardiac events; MI = myocardial infarction; MLD = minimal luminal diameter; PCI = percutaneous coronary intervention.

Following repeat PCI, 2 of the 6 patients with mid-term MACE were later documented to be free from angina. As of the last known follow-up (16.8 ± 14.1 months), nineteen of 22 patients (87%) were alive and free from angina. Patients were more likely to be alive and free from angina if they had a protected LMCA (94% vs. 57% with unprotected LMCA; P = 0.0564) and if they received a DES (89% vs. 50% with no stent; P = 0.0281). Angiographic follow-up was available in 9 of 21 (43%) patients with DESs at a mean of 13.7 ± 11.8 months revealing a mean late loss of 0.27 ± 0.45 mm and binary restenosis in 1 of 9 (11%) (Table 3).

## Discussion

This study sought to describe the contemporary use of RA in the LMCA to facilitate DES placement. The main outcomes of this analysis suggest that RA in the LMCA is safe and feasible and facilitates DES implantation with high procedural success. Compared with RA alone in the LMCA, RA and DES implantation is associated with a higher angiographic success rate. Patients were more likely to be alive and free from angina at final follow-up if they had a protected LMCA and received a DES.

The benefits of RA have previously been described and include improving angiographic outcomes and facilitating stent implantation in heavily calcified lesions [7-10, 21, 22] and preserving the patency of side branches in bifurcation and ostial lesions [12, 13]. In heavily calcified lesions, RA combined with a DES resulted in target lesion revascularization rates ranging from 2% to 10.6% at 6 months to 3 years of follow-up [14-18]. As these reports focused on non-LMCA PCI, we sought to extend these concepts to the LMCA. These concepts are particularly relevant to LMCA lesions which often become symptomatic at the later stages of coronary artery disease, frequently years after prior CABG surgery, involve the distal bifurcation over 60% of the time and frequently contain severe calcification [2, 4, 5].

The results of this contemporary series of 31 patients with LMCA lesions treated with RA suggest that the previous findings of RA in the non-LMCAs translate to the LMCA. A DES was successfully implanted in all patients without a contraindication to DESs, despite severe vessel calcification. There were no procedural complications. One major adverse cardiovascular event (3%) occurred prior to hospital discharge: a 97-year-old woman with an unprotected LMCA and multiple comorbidities died of pneumonia. The procedure was well tolerated by each patient and acute morbidity was minimal in this patient cohort with high-risk LMCA disease. Even in patients with unprotected LMCAs, RA was used in conjunction with left ventricular assist devices without procedural complications.

At last known follow-up (16.8 ± 14.1 months), patients were more likely to be free from angina if they had a protected LMCA and received a DES. MACE tended to occur less frequently in patients with protected versus unprotected LMCAs. These results suggest that patients with protected LMCAs have favorable mid-term outcomes following RA with a DES. Patients with unprotected LMCAs can safely undergo RA, but data on mid-term outcomes is inconclusive.

This series is compatible with other reports on patients undergoing LMCA interventions. Death and MACE are known to occur more frequently in patients undergoing PCI for unprotected LMCA disease compared with protected LMCA disease [23]. Patients undergoing LMCA PCI have better long-term outcomes with DES implantation compared with no stent or even with bare metal stents [2]. Improved long-term outcomes in patients treated with DESs compared with no stent were not observed in our series, probably due to a small sample size and limited patient follow-up. Death and MACE rates (at about 15 months) were slightly higher for this entire cohort (death 13%, MACE 26%), DES group (11%, 22%), and unprotected LMCA group (29%, 43%) compared with other reports of 12 month outcomes for patients with LMCA disease undergoing DES implantation (∼5%, ∼10-15%) or CABG (∼10%, ∼23%) (beyond 12 months outcomes favor CABG compared with DES) [2]. The increase in death and MACE at 1 year observed in this series may be due to small sample size, incomplete patient follow-up, and most importantly selection bias. Unlike many reports with inclusion and exclusion criteria that exclude the most critically ill patients, this series, by selecting only patients for which RA was deemed necessary, selected only patients with severe coronary artery disease (severe calcification) and comorbid conditions (including myocardial infarction presentation and 1 patient in cardiogenic shock).

Initial reports of RA described relatively high complication rates including vessel dissection in the range of 5-10% and perforation necessitating emergent CABG surgery in approximately 1% [22, 24-26]. However, when RA is employed as a stand alone procedure, as in the initial reports, a relatively large burr-to-vessel ratio (> 0.7) was used to achieve optimal rotablation [24-26]. When RA is employed to facilitate stent implantation, smaller burr-to-vessel ratios are commonly used, ranging from 0.5 - 0.6 [9, 11, 15, 27]. In this cohort of LMCA lesions a final burr-to-vessel ratio of 0.43 was used. When RA is employed to facilitate stent expansion, optimal rotablation is not necessary, but rather the goal is to change the compliance of the calcified vessel so that optimal stent expansion can be achieved. By reducing the rigidity and eccentricity of a heavily calcified lesion, stent expansion is optimized and more symmetric [10]. As the LMCA exhibits substantial elastic recoil [19], balloon angioplasty does not contribute significantly to acute gain in the LMCA; most of the initial acute gain can be attributed to RA rather than balloon angioplasty. The benefit of RA, however, cannot be measured by its immediate acute gain, but rather by the final acute gain after stenting which could not be realized without adjuvant RA. Similarly, relatively smaller burr-to-vessel ratios are needed to debulk plaque burden and prevent side branch occlusion [13]. Adjunctive rotablation may have additional benefits when used with DESs by limiting trauma to the stent coating and improving drug delivery to the subintimal tissue [15].

The findings in the present report support the utility of RA in which a smaller burr-to-vessel ratio is used for heavily calcified lesions of the LMCA. The goal of RA in this setting is to facilitate stent implantation and to prevent occlusion of the left anterior descending and left circumflex coronary arteries when the distal bifurcation is involved. RA was used successfully as a “bail-out” procedure after the balloon catheter could not be delivered to a protected LMCA lesion in 2 patients. The use of RA probably spared these 2 patients from a repeat CABG surgery.

Left ventricular assist devices (mainly TandemHeart) were used in patients with depressed left ventricular function undergoing PCI on an unprotected LMCA lesion. The American College of Cardiology/American Heart Association guidelines recommend cardiac assist devices in such high-risk interventions [3].

The relatively small initial percent diameter stenosis is related to our use of the proximal LMCA to define reference vessel diameter, while most lesions involved the distal segment. Also, several lesions spanned both the LMCA and one of its branches with the true MLD distal to the bifurcation of the LMCA. For this report on LMCA interventions, QCA measurements were confined to the LMCA.

There are several limitations of the present study. As a retrospective analysis at a single center it is uncertain whether these results will translate prospectively to clinical practice in the community. This analysis included a small number of patients; they may not be representative of larger patient populations. Follow-up data was only available for patients who returned to our hospital to be evaluated by our physicians (23 of 31 patients; 74%), and was limited to only a few months for several patients. The majority of patients with limited or no follow-up were referred from their primary cardiologist for a complex PCI, then returned to their primary cardiologist for follow-up care. Some late major adverse cardiac events may have been missed.

### Conclusion

In summary, RA of the LMCA is safe and feasible and facilitates DES implantation with high procedural success. Favorable mid-term outcomes were observed following RA and DES implantation in heavily calcified, protected LMCAs. The results suggest that routine use of RA prior to DES implantation may be considered in patients with heavily calcified lesions involving the distal LMCA.
